# Case Report: Longitudinal mass cytometry profiling of a patient with disseminated histoplasmosis and secondary hemophagocytic lymphohistiocytosis

**DOI:** 10.3389/fimmu.2025.1660382

**Published:** 2025-10-01

**Authors:** Jiaying Zhang, Longyu Zhang, Zixin Kang, Danlei Mou, Lianchun Liang, Yu Chen, Yingmei Feng

**Affiliations:** ^1^ Department of Infectious Diseases, Beijing Youan Hospital, Capital Medical University, Beijing, China; ^2^ Laboratory for Clinical Medicine, Capital Medical University, Beijing, China; ^3^ Fourth Department of Liver Disease, Beijing Youan Hospital, Capital Medical University, Beijing, China; ^4^ Department of Science and Development, Beijing Youan Hospital, Capital Medical University, Beijing, China

**Keywords:** histoplasmosis, hemophagocytic lymphohistiocytosis, mass cytometry, immunemetabolism, cytokine storm

## Abstract

Disseminated histoplasmosis (DH) is a rare but serious systemic fungal infection that can trigger secondary hemophagocytic lymphohistiocytosis (HLH), a hyperinflammatory syndrome with high mortality. However, the immunopathogenesis of DH-associated HLH remains poorly defined due to the lack of high-resolution immune profiling data. The dynamics of immunological and metabolic analysis was performed in a 14-year-old female patient with DH-HLH using mass cytometry (CyTOF) and multiplex cytokine profiling. Peripheral blood mononuclear cells and plasma were collected at three timepoints: before antifungal treatment, and at 1, and 2 weeks post-treatment, respectively. Immune subsets, functional markers, and cytokine/chemokine levels were evaluated. Mass cytometry identified 13 distinct immune cell subsets, including NK cells, double-negative T (DNT) cells, memory CD8^+^ T cells, and M2 macrophages. Longitudinal analysis demonstrated a progressive decline in proinflammatory cytokines (such as IL-6, TNF-α, and IP-10) accompanied by an expansion of reparative subsets, particularly M2 macrophages. Concurrent immune-metabolic profiling revealed a metabolic shift from glycolysis to lipid oxidation, characterized by decreased expression of GLUT1 and CPT1A and increased expression of CD36. This transition from a glycolysis-driven inflammatory state to an oxidative, immunoregulatory phenotype correlated with clinical recovery and attenuation of the cytokine storm. This case demonstrates the utility of mass cytometry for dynamic immune monitoring in infection-triggered HLH. The findings highlight metabolic reprogramming and immune restoration as key features of disease resolution and suggest potential immunometabolic targets for future diagnostic and therapeutic strategies.

## 1 Introduction

Histoplasmosis is a systemic fungal infection caused by *Histoplasma capsulatum*, primarily transmitted through the inhalation of spores from contaminated environments. The disease predominantly affects the lungs and may be asymptomatic or present with flu-like symptoms in mild cases. However, in immunocompromised individuals, it can progress to disseminated histoplasmosis (DH), involving multiple organ systems such as the liver, spleen, bone marrow, and central nervous system (1, 2). Between 2012 and 2022, a total of 225 cases of histoplasmosis were reported in China, with an increasing trend in incidence observed over the past 11 years (2, 3). Hemophagocytic lymphohistiocytosis (HLH) is a severe disorder of immune dysregulation characterized by excessive immune activation and an overwhelming inflammatory response. HLH is classified into primary (familial HLH) and secondary (acquired HLH) forms. Secondary HLH can be triggered by infections, malignancies, or autoimmune diseases (4, 5).

Histoplasmosis can serve as a potential trigger for HLH, particularly in immunocompromised individuals, including those with HIV infection, recipients of solid organ transplants, patients with hematologic malignancies, or those receiving long-term immunosuppressive therapy (6–9). Disseminated histoplasmosis may induce robust immune activation, leading to abnormal macrophage proliferation and phagocytosis of blood cells, thereby precipitating HLH (10, 11). Moreover, the occurrence of HLH may further exacerbate the course of histoplasmosis, making the infection more difficult to control. While HLH is characterized by immune hyperactivation and uncontrolled macrophage proliferation, the immunopathogenesis of DH-triggered HLH remains poorly understood due to limited mechanistic studies. Currently, most immunological assessments in HLH rely on serum cytokine levels and conventional flow cytometry. However, these approaches fall short in capturing the complexity of cellular immune responses. Mass cytometry (CyTOF) has been successfully applied in oncology, autoimmunity, and viral infections to map immune dysregulation and identify novel cellular subsets and therapeutic targets (12, 13). Despite this, CyTOF has not yet been systematically explored in the setting of fungal infections or HLH. Given its ability to comprehensively profile immune cell phenotypes and signaling pathways at single-cell resolution, CyTOF holds promise for identifying novel diagnostic biomarkers and therapeutic targets in patients with disseminated histoplasmosis-associated HLH.

In this study, we present a rare case of DH-HLH and apply mass cytometry combined with expression profiling to map immune dysfunction in detail. Our aim is to highlight the diagnostic and mechanistic value of high-dimensional immunophenotyping in this underexplored clinical context.

## 2 Case presentation

A 14-year-old girl presented with a six-month history of intermittent high-grade fever (up to 40°C), accompanied by chills, rigors, fatigue, alopecia, and a weight loss of 7.5 kg. She denied respiratory, gastrointestinal, or neurological symptoms. Multiple prior empirical antibiotic treatments were ineffective.

On admission, her vital signs showed a temperature of 40°C, heart rate 96 bpm, and blood pressure 110/60 mmHg. She appeared fatigued but was alert and developmentally appropriate. Physical examination revealed pallor, cervical lymphadenopathy (0.5 × 0.5 cm, non-tender), hepatomegaly (liver edge at umbilicus), splenomegaly (4 fingerbreadths below the left costal margin), and mild bilateral lower limb oedema.

**Figure 1:**
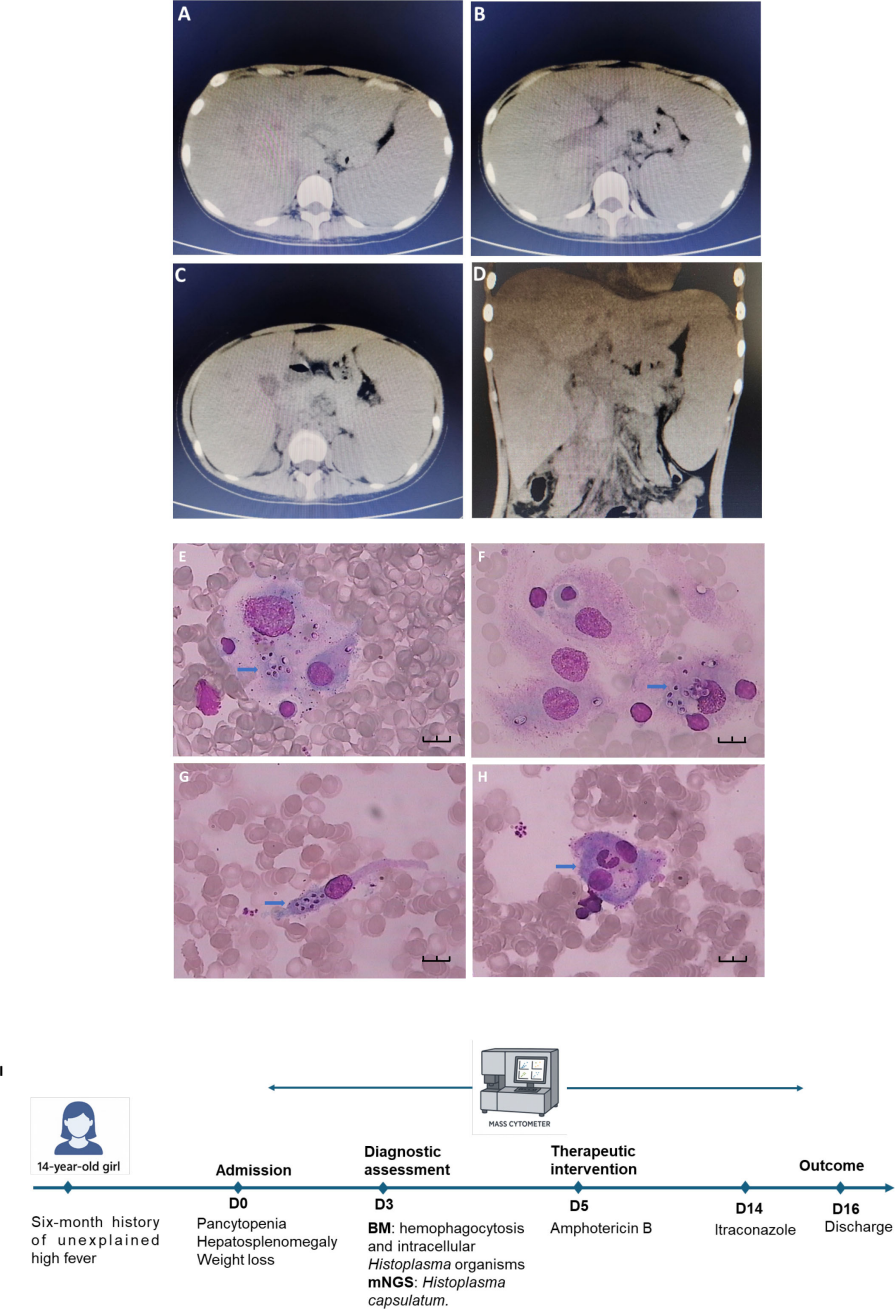
Abdominal computed tomography (CT), bone marrow smear and clinical timeline of the patient. **(A–C)** Axial CT images demonstrate hepatosplenomegaly with multiple hypodense lesions in the liver and spleen, suggestive of disseminated infectious infiltration. **(D)** Coronal view shows irregular thickening of the intestinal wall and enlarged mesenteric lymph nodes, indicative of systemic inflammatory response. Bone marrow smear were stained with Wright-Giemsa and examined under oil immersion (×1000). Black scale bars represent 10 μm. **(E–G)** display macrophages containing intracellular Histoplasma organisms (blue arrows). The fungal cells appear round to ovoid (2–4 μm), with pale blue cytoplasm, a centrally located basophilic nucleus-like structure, and a surrounding clear halo. **(H)** highlights an enlarged histiocyte (blue arrow) with abundant pale violet cytoplasm and well-defined borders. Phagocytosed neutrophils are visible within the cytoplasm, indicative of hemophagocytosis. **(I)** Timeline of the patient’s clinical course. BM, bone marrow. mNGS, metagenomic Next-Generation Sequencing.

Laboratory tests showed pancytopenia: WBC 1.38 × 10^9^/L, Hb 84 g/L, PLT 52 × 10^9^/L. Albumin was low (26 g/L), while liver enzymes and renal function were normal. Inflammatory markers were elevated: CRP 60.12 mg/L, PCT 5.76 ng/mL, ferritin 635 ng/mL, and soluble CD25 at a markedly high level of 52,992 pg/mL. The (1→3)-β-D-glucan (G test) was positive (204.4 pg/mL); GM test was negative. Serologic tests for HIV, syphilis, hepatitis B, tuberculosis (T-SPOT.TB), and Widal were negative. EBV-DNA and CMV-DNA were undetectable. Immunophenotyping revealed decreased CD4^+^ T cells (92/μL) and impaired NK cell activity (12.79%). Autoimmune screening was negative, except for a weakly positive direct antiglobulin test. Imaging studies included chest CT showing mild bilateral pulmonary infiltrates and pleural effusion. Abdominal CT revealed hepatosplenomegaly, multiple enlarged abdominal and retroperitoneal lymph nodes, and ascites (**Figures 1A–D**).

The constellation of prolonged fever, hepatosplenomegaly, pancytopenia, hyperferritinemia, elevated sCD25, and reduced NK cell activity met diagnostic criteria for HLH. Bone marrow aspiration showed hypocellularity with prominent hemophagocytosis and macrophage aggregation. Importantly, numerous intracellular yeast-like organisms suggestive of *Histoplasma* were identified morphologically (**Figures 1E–H**).

Metagenomic next-generation sequencing (mNGS) of bone marrow confirmed *Histoplasma capsulatum* (17 sequence reads), leading to a diagnosis of DH-associated HLH. The patient was treated with intravenous amphotericin B. Within five days, her fever subsided, and inflammatory markers significantly improved. After completing intravenous amphotericin B, she was placed on oral itraconazole for consolidation therapy and discharged in stable condition (**Figure 1I**).

## 3 Literature review of histoplasmosis cases complicated by HLH

Our literature review identified a total of 71 reported cases of histoplasmosis complicated by HLH, among which 51 patients survived and 20 died, resulting in an overall case fatality rate of 28.17% (**Supplementary Tables S1**, **S2**). These cases remain exceedingly rare, and to date, no published studies have provided longitudinal immune profiling using single-cell or cytokine-based analyses in this context.

## 4 Longitudinal mass cytometry profiling

**Figure 2:**
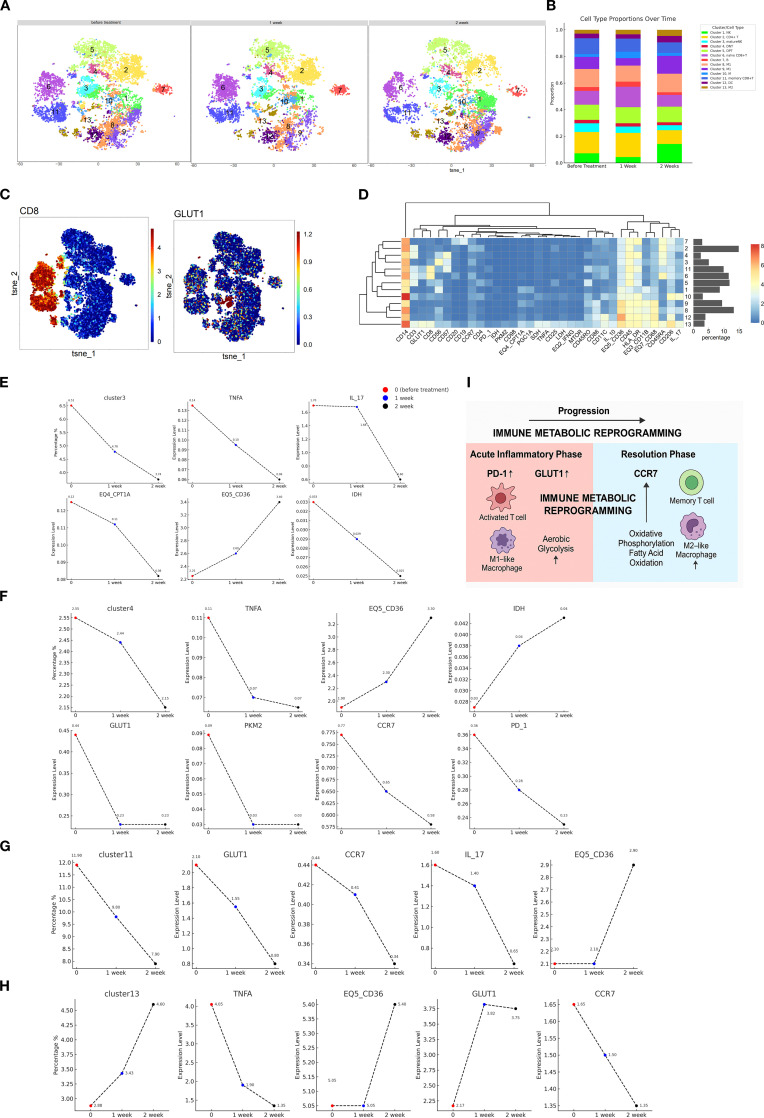
Single-cell landscape of immune cell distribution, functional marker expression, and temporal dynamics. **(A)** t-SNE–based dimensionality reduction was used to visualize peripheral immune cell clustering at baseline, week 1, and week 2. Each color represents a distinct cell cluster. **(B)** The bar graph on the right illustrates the relative proportions of immune subsets over time, revealing immune remodeling after treatment with notable expansion or contraction of specific populations. **(C)** Spatial distribution of key functional markers overlaid on the t-SNE map. CD8 identify T cells and their subsets, while GLUT1, a glucose transporter, reflects cellular metabolic activation. Heatmap intensity ranges from low (blue) to high (red), indicating heterogeneous marker expression across clusters and dynamic functional states during treatment. **(D)** Cluster-specific phenotypic heatmap. The x-axis displays major surface and functional markers (e.g., CD14, CD3, GLUT1, HLA-DR, PD-1, TIGIT), and the y-axis shows clusters (Cluster 0–13) derived from t-SNE–based analysis. Heatmap color indicates average marker expression per cluster (blue: low, red: high), reflecting distinct phenotypic and functional profiles. The adjacent bar plot presents the relative abundance (%) of each cluster across samples. **(E–H)** Dynamic trends of immune subsets and marker expression during treatment. Temporal trajectories of cell frequency and phenotypic marker expression for clusters 3, 4, 11, and 13 at baseline (red), week 1 (blue), and week 2 (black). **(I)** Dynamic immune remodeling during antifungal therapy. Longitudinal changes in immunometabolic profiles during antifungal therapy in a patient with DH-associated HLH. Single-cell CyTOF and multiplex cytokine profiling revealed dynamic shifts in immune cell subsets, inflammatory mediators, and metabolic markers over the course of treatment.

To track the dynamic changes in immune cell subsets and their functional marker expression in the patient, PBMCs collected at baseline, week 1, and week 2 were analyzed using mass cytometry. A concise summary of the CyTOF analysis workflow is provided here, while detailed information on the antibody panel, staining procedures, data acquisition, and analysis pipelines is described in **Supplementary Material S2**. CD45^+^ immune cells were gated, and unsupervised clustering identified 13 distinct immune cell subsets based on differential expression of lineage and functional markers (**Figure 2A**). These subsets were annotated as follows: cluster 1, natural killer (NK) cells; cluster 2, CD4^+^ T cells; cluster 3, mature NK cells; cluster 4, double-negative T (DNT) cells; cluster 5, double-positive T (DPT) cells; cluster 6, naïve CD8^+^ T cells; cluster 7, B cells; cluster 8, M1 macrophages; cluster 9, lipid metabolism–enriched M1 macrophages; cluster 10, monocytes; cluster 11, memory CD8^+^ T cells; cluster 12, dendritic cells; and cluster 13, M2 macrophages (**Figure 2B**). The relative frequencies of these 13 clusters across the three time points are presented in **Table 1**. Representative immune cell subsets and their associated molecular signatures are illustrated in **Figures 2C, D**, which highlight dynamic changes in representative immune cell subsets and the expression profiles of key functional proteins.

**Table 1:** Summary of clusters in PBMC identified by mass cytometry.

Cluster/cell subpopulation	Before treatment	1 week	2 week
Cluster 1, NK cells	7.25	4.3875	14.25
Cluster 2, CD4^+^ T cells	15.9625	18.125	10.325
Cluster 3, Mature NK cells	6.5125	4.775	3.7375
Cluster 4, Double-negative T (DNT) cells	2.55	2.4375	2.15
Cluster 5, Double-positive T (DPT) cells	11.4625	12.1375	11.8125
Cluster 6, Nnaïve CD8^+^ T cells	10.35	15.4125	8.8125
Cluster 7, B cells	2.875	3.75	1.9375
Cluster 8, M1 macrophages	13.6	12.075	13.975
Cluster 9, Lipid-enriched M1 macrophages	9.1	5.6	13.375
Cluster 10, Monocytes	2.0625	4.8	2.2375
Cluster 11, Memory CD8+ T cells	11.925	9.7875	7.85
Cluster 12, Dendritic cells	3.475	3.2875	4.9375
Cluster 13, M2 macrophages	2.875	3.425	4.6

The data are expressed as percentages, indicating the distribution of immune cell clusters in the patient before treatment, and at 1 and 2 weeks following antifungal therapy.

As illustrated in **Figure 2E**, cluster 3 was annotated as mature natural killer (NK) cells, comprising 3.74% to 6.51% of total CD45^+^ immune cells. The relatively low abundance of mature NK cells suggests a functional impairment, which may be mechanistically linked to the immunopathogenesis of HLH. Notably, the expression levels of pro-inflammatory cytokines TNF-α and IL-17 within NK cells exhibited a decreasing trend, indicating a progressive attenuation of the hyperinflammatory state. The expression of CD36 (EQ5_CD36), involved in fatty acid uptake and lipid signaling, remained elevated during treatment. Upregulation of CD36 may reflect the reprogramming of lipid metabolic pathways, supporting NK cell functional remodeling and sustaining long-term immunosurveillance. In contrast, expression of CPT1A (EQ4_CPT1A), a rate-limiting enzyme in fatty acid β-oxidation, progressively declined after treatment. This trend suggests that NK cells may transition into a metabolically quiescent state. A gradual reduction in the expression of isocitrate dehydrogenase (IDH) was observed post-treatment. This pattern suggests a metabolic transition from glycolysis to a more oxidative phenotype dominated by the tricarboxylic acid (TCA) cycle.

As shown in **Figure 2F**, cluster 4 was identified as DNT cells. An increased proportion of DNT cells is commonly observed in HLH, possibly reflecting a persistently activated immune state. In this study, the percentage of DNT cells progressively declined following treatment, suggesting a gradual restoration of immune homeostasis. Functionally, the expression of tumor necrosis factor-α (TNF-α) in DNT cells showed a decreasing trend after treatment, indicative of reduced inflammatory signaling. Expression of programmed cell death protein 1 (PD-1), a marker of T cell exhaustion and immune suppression, also declined over time, implying a relief of immunoinhibitory pressure as treatment progressed. Similarly, C-C chemokine receptor type 7 (CCR7), which mediates lymphocyte trafficking and homing, showed a downward trend, suggesting altered migratory dynamics of lymphocytes during immune recovery. Glucose transporter 1 (GLUT1), a key mediator of glucose uptake, was significantly downregulated after treatment, indicating reduced glycolytic activity in DNT cells. This may reflect a metabolic transition from glycolysis to a more stable, oxidative energy profile. Pyruvate kinase M2 (PKM2), a rate-limiting enzyme in the glycolytic pathway and a marker of T cell activation, was also markedly decreased, consistent with attenuated T cell activation and reduced inflammatory metabolism. Expression trends of IDH and CD36 in DNT cells mirrored those observed in NK cells, suggesting similar metabolic reprogramming processes underlying immune resolution.

As shown in **Figure 2G**, cluster 11 was annotated as memory CD8^+^ T cells, accounting for 7.85% to 11.93% of total immune cells. These cells exhibited a downward trend in IL-17 expression over the course of treatment, suggesting a reduction in proinflammatory activity. Metabolically, GLUT1, a key facilitator of glycolytic flux, showed decreased expression after treatment, indicating reduced glucose uptake and a possible shift away from glycolysis. In contrast, CD36 (EQ5_CD36), a key mediator of lipid uptake and metabolic reprogramming, was upregulated following treatment, suggesting a shift toward enhanced lipid metabolism that may facilitate the survival and functional persistence of memory CD8^+^ T cells. Furthermore, CCR7, a chemokine receptor associated with lymphocyte trafficking and immune regulation, demonstrated a progressive decline in expression post-treatment, which may reflect altered migratory behavior and tissue retention of memory CD8^+^ T cells during immune reconstitution.

As shown in **Figure 2H**, cluster 13 was identified as M2 macrophages, comprising 2.88% to 4.60% of total immune cells. Expression of TNF-α demonstrated a decreasing trend during treatment, suggesting attenuation of inflammatory activity. At the metabolic level, both CD36 and GLUT1, markers associated with lipid uptake and glucose transport respectively, showed progressive downregulation following treatment. This pattern may indicate a shift toward a metabolically quiescent or anti-inflammatory phenotype. Additionally, CCR7 also declined over time, suggesting a reduced migratory capacity or altered tissue localization of M2 macrophages during immune recovery.

Peripheral blood cytokine profiling revealed substantial immune dysregulation in the patient (**Table 2**). Compared with healthy controls, the patient exhibited markedly elevated levels of several proinflammatory cytokines and chemokines, including Eotaxin, G-CSF, GRO, IL-1α, IL-6, IL-8, IP-10, MCP-1, MCP-3, MIG, MIP-1α, MIP-1β, and TNF-α. Conversely, cytokines associated with tissue regeneration and immune modulation, such as sCD40L, EGF, FGF-2, FLT-3L, IL-3, IL-5, IL-7, IL-9, IL-12(p40), IL-13, IL-15, PDGF-AA, TGF-α, and VEGF, were notably decreased. During the course of treatment, levels of Eotaxin, G-CSF, IL-6, IL-8, IP-10, MCP-1, and MIG gradually declined, indicating a resolution of systemic inflammation and the initiation of tissue repair processes. Immune profiling by CyTOF and cytokine analysis across clinical stages revealed dynamic changes following therapy (**Figure 2I**).

**Table 2:** Immune and inflammatory cytokine profiles.

Cytokine	Healthy controls	1 week	2 week
sCD40L (pg/mL)	979.4 (672.3, 1286.5)	529.15	585.15
EGF (pg/mL)	39.7 (28.1, 51.3)	36.69	44.86
Eotaxin (pg/mL)	47.5 (41.3, 53.8)	67.94	53.50
FGF-2 (pg/mL)	74.0 (56.1, 91.9)	67.16	63.34
FLT-3L (pg/mL)	10.8 (8.7, 13.0)	7.94	3.28
Fractalkine (pg/mL)	97.1 (76.4, 117.9)	315.50	251.60
G-CSF (pg/mL)	18.9 (14.2, 23.5)	69.01	46.15
GRO-α (pg/mL)	15.5 (10.8, 20.2)	25.84	26.50
IFN-α2 (pg/mL)	39.7 (26.5, 53.0)	163.55	120.95
IFN-ϒ (pg/mL)	2.1 (0.9, 3.2)	4.37	4.12
IL-1α (pg/mL)	6.9 (4.5, 9.2)	33.28	18.36
IL-1β (pg/mL)	8.5 (5.2, 11.8)	10.29	6.65
IL-1RA (pg/mL)	9.7 (6.6, 12.8)	17.44	13.91
IL-2 (pg/mL)	0.6 (0.3, 0.8)	1.94	1.63
IL-4 (pg/mL)	1.2 (0.8, 1.5)	3.37	2.06
IL-5 (pg/mL)	3.4 (2.6, 4.3)	2.66	1.73
IL-6 (pg/mL)	1.1 (0.4, 1.9)	7.91	8.97
IL-7 (pg/mL)	2.3 (1.8, 2.9)	1.83	1.73
IL-8 (pg/mL)	3.1 (1.9, 4.4)	5.71	4.44
IL-9 (pg/mL)	6.5 (3.6, 9.3)	38.58	34.02
IL-12 (p40) (pg/mL)	40.0 (29.4, 50.6)	489.92	454.32
IL-12 (p70) (pg/mL)	3.3 (2.4, 4.1)	14.81	14.43
IL-13 (pg/mL)	32.7 (24.1, 41.4)	81.14	60.00
IL-15 (pg/mL)	5.9 (4.6, 7.3)	10.92	6.85
IL-17A (pg/mL)	4.2 (2.4, 6.0)	13.61	12.31
IL-17E/IL-25 (pg/mL)	308.6 (224.1, 393.2)	797.46	604.74
IL-18 (pg/mL)	68.7 (40.3, 97.0)	132.29	57.69
IL-27 (pg/mL)	768.4 (660.3, 876.5)	2184.00	1926.00
IP-10 (pg/mL)	133.9 (105.5, 162.3)	1683.00	1125.00
MCP-1 (pg/mL)	146.0 (130.6, 161.4)	335.44	248.06
MCP-3 (pg/mL)	18.8 (16.1, 21.4)	39.00	28.91
M-CSF (pg/mL)	38.4 (29.3, 47.5)	232.57	210.32
MDC (pg/mL)	361.0 (310.9, 411.2)	568.80	540.90
MIG (pg/mL)	529.2 (448.9, 609.4)	31057.00	22783.00
MIP-1α (pg/mL)	22.0 (16.9, 27.1)	48.99	39.07
MIP-1β (pg/mL)	15.3 (13.0, 17.5)	60.95	57.96
PDGF-AA (pg/mL)	1117.9 (912.0, 1323.9)	1325.00	1683.00
PDGF-AB/BB (pg/mL)	19435.3 (16659.3, 22211.3)	3450.00	4608.00
RANTES (pg/mL)	2952.0 (2449.5, 3454.5)	1881.00	1618.00
TGF-α (pg/mL)	1.8 (1.5, 2.1)	5.18	4.37
TNFα (pg/mL)	20.0 (17.1, 22.9)	152.15	115.38
TNFβ (pg/mL)	6.8 (4.7, 8.9)	10.03	8.41
VEGF-A (pg/mL)	54.3 (33.9, 74.7)	30.10	25.88

Cytokine levels are shown as means (95% confidence intervals) along with actual measured values. The data represent the reference range derived from 84 healthy individuals, and the patient’s values at 1 and 2 weeks post-treatment.

## 5 Discussion

Using longitudinal mass-cytometry and multiplex cytokine profiling, we tracked the immunometabolic landscape of a patient with DH complicated by HLH. HLH is driven by uncontrolled activation of cytotoxic lymphocytes and macrophages, culminating in a cytokine storm and multi-organ injury (5). The single-cell resolution afforded by CyTOF uncovered 13 phenotypically distinct immune subsets and revealed their stepwise re-equilibration within two weeks of antifungal therapy, illustrating how immune networks re-wire as hyper-inflammation subsides. We systematically profiled the patient’s immune status using CyTOF and multiplex cytokine analysis across distinct clinical timepoints, from the acute phase to recovery. The dynamic changes observed before and after antifungal therapy are illustrated in **Figure 2**. To our knowledge, this is one of the very few reports in the past two decades that combines immunophenotypic and molecular analyses in a pediatric patient with DH-associated HLH. Our findings therefore offer novel insights into the immunopathogenesis and therapeutic response of this rare but life-threatening condition.

In hyperinflammatory conditions such as HLH and severe infections, T cells often undergo excessive activation, resulting in the massive secretion of pro-inflammatory cytokines such as interferon-γ and IL-6, which lead to cytokine storm (14). This is consistent with the characteristic cytokine profile observed in HLH, as summarized by Wu et al. (15), who also highlighted Epstein-Barr virus (EBV) as a common infectious trigger of secondary HLH. This pathologic immune overactivation exacerbates tissue damage and establishes a positive feedback loop among immune cells, ultimately resulting in multiple organ dysfunction (16). Compared with healthy controls, the patient displayed a prototypical HLH cytokine storm: IL-6, IL-8, TNF-α, MCP-1, IP-10 and related chemokines were markedly elevated, whereas regenerative mediators such as EGF, FLT-3L and VEGF were suppressed. This pattern mirrors previous studies on infection-associated HLH (15, 17). Following antifungal therapy, there was a marked decline in pro-inflammatory cells and cytokines in the patient’s peripheral blood, including NK cells, subsets of activated T cells, and inflammatory mediators such as TNF-α and IL-1β. Concurrently, an increase was observed in M2 macrophages and regenerative growth factors such as PDGF-AA and PDGF-AB/BB, indicating a gradual transition from a hyperinflammatory state to a more homeostatic or tissue-reparative immune profile. Moreover, targeted immunomodulatory therapies, including those directed at the JAK/STAT signaling pathway or specific cytokines like IL-6, have demonstrated efficacy in interrupting the pathogenic interplay between T cells and macrophages, highlighting the therapeutic potential of precise immune intervention (15, 18, 19).

In addition to cytokine dysregulation, the patient’s laboratory results revealed classic features of HLH-associated cytopenia. Complete blood counts demonstrated profound pancytopenia, with WBC reduced to 1.38 ×10^9^/L, neutrophils at 0.16 ×10^9^/L, lymphocytes at 0.16 ×10^9^/L, and monocytes at 0.05 ×10^9^/L, well below the lower limits of normal. Platelet counts were also markedly decreased (PLT 52 ×10^9^/L), and hemoglobin was moderately reduced (Hb 84 g/L). Differential leukocyte analysis further supported an immunosuppressed state with severe depletion across both myeloid and lymphoid compartments. Notably, flow cytometric immunophenotyping revealed an absolute CD4^+^ T cell count <92/μl, indicative of severe T cell depletion. These findings highlight the coexistence of systemic hyperinflammation and immune exhaustion, consistent with the dual-phase immunopathology often seen in secondary HLH (18, 20). The incorporation of these laboratory data strengthens the interpretation of CyTOF and cytokine results and provides a more comprehensive picture of the patient’s immune dysfunction and trajectory of recovery.

NK cells, which are crucial for immune regulation and cytotoxic defense, are frequently found to be depleted or functionally impaired due to persistent antigenic stimulation. Studies have consistently shown that both primary and secondary HLH are associated with significantly reduced NK cell cytotoxic activity, which correlates closely with disease severity and prognosis (5, 15). Sustained immune overactivation may lead to marked depletion of NK cells or significant impairment of their cytotoxic function. Aberrant expansion of DNT cells has also been observed in certain autoimmune diseases and chronic infections. In this case, antifungal therapy targeting *Histoplasma capsulatum* effectively suppressed the overactivated T cell subsets. As the disease came under control, the proportion of DNT cells declined, suggesting a resolution of the aberrant T cell proliferation. In disseminated histoplasmosis, persistent exposure to fungal antigens may drive chronic T cell activation. Once antifungal treatment reduces the fungal burden, the diminished antigenic stimulation may lead to a contraction of memory CD8^+^ T cells, which can no longer be maintained in the absence of ongoing stimulation.

In the later stages of therapy, the immune system undergoes reorganization: as chronic inflammation resolves, the memory T cell pool contracts and stabilizes at a new homeostatic level, reflective of a recalibrated immune equilibrium (21, 22). M2 macrophages are generally regarded as “anti-inflammatory” or “immunosuppressive” in phenotype, and their numbers often increase during chronic inflammation or specific infectious states to facilitate pathogen clearance and promote tissue repair (23). In conditions such as HLH and severe infections, an initial surge in pro-inflammatory cells, including M1 macrophages and activated monocytes, and cytokines often leads to a cytokine storm (24). As treatment becomes effective and the hyperinflammatory response is gradually brought under control, the proportion of M2 macrophages typically rises. This shift indicates a transition toward an immunosuppressive or tissue-reparative state as in **Figure 2I**, reflecting a change in the immune landscape during recovery (21).

It is particularly noteworthy to consider the changes observed in immune regulatory molecules such as PD-1 and CCR7. PD-1 is a key inhibitory receptor expressed on T cells, and its sustained upregulation is widely recognized as a hallmark of T cell exhaustion, often occurring in chronic infections, cancer, and prolonged inflammation (25, 26). High PD-1 expression suggests a dysfunctional immune state characterized by impaired cytokine secretion, reduced proliferative capacity, and weakened cytotoxic function (27). In contrast, CCR7 plays a pivotal role in lymphocyte homing to secondary lymphoid organs and is essential for the maintenance and functionality of central memory T cells (25). Clinically, patients subjected to persistent inflammatory stimuli or immunosuppressive therapies, such as glucocorticoids, frequently exhibit a concurrent increase in PD-1 and decrease in CCR7 expression in peripheral blood T cells, indicating compromised T cell activity and impaired migratory capacity (28). This immunophenotype raises concerns regarding the emergence of T cell exhaustion and a breakdown in immune surveillance, ultimately predisposing patients to secondary infections and delayed immune recovery (29). Therefore, beyond merely controlling hyperinflammation, therapeutic strategies should also include close monitoring of immune suppression markers and early intervention to restore T cell function when signs of exhaustion become apparent.

In addition, dynamic changes in key metabolic regulators such as glucose transporter 1 (GLUT1), detected by mass cytometry-based protein profiling, further highlight the immunometabolic reprogramming that occurred over the disease course. GLUT1 is a hallmark of glycolytic activation and closely associated with the Warburg effect, a metabolic shift whereby immune cells, particularly activated T cells and M1 macrophages, prefer aerobic glycolysis over oxidative phosphorylation (OXPHOS) to rapidly generate ATP and biosynthetic intermediates during periods of high inflammatory stress and proliferative demand (30–32). In this case, elevated GLUT1 expression during the acute phase suggests an active Warburg-like metabolic state. However, following therapeutic intervention, GLUT1 and other glycolysis-related proteins were found to be downregulated, implying a reduction in systemic inflammation and cellular proliferation. Concurrently, a rise in M2 macrophages and immune subsets associated with suppression and repair indicated a phenotypic and metabolic shift toward fatty acid oxidation (FAO) and mitochondrial OXPHOS. Similarly, isocitrate dehydrogenase (IDH), particularly the mitochondrial IDH3 isoform, modulates tricarboxylic acid (TCA) cycle flux and redox homeostasis. Through regulation of α-ketoglutarate levels and HIF-1α stabilization, IDH activity plays a crucial role in determining the balance between glycolysis and OXPHOS (33). Together, these findings underscore a progressive shift in immunometabolism from a glycolytic, pro-inflammatory profile toward an oxidative, reparative phenotype, mirroring the patient’s transition from acute inflammation to immune resolution and tissue recovery.

This study has several limitations. Although longitudinal samples were analyzed, the investigation was conducted on a single patient, which limits the generalizability of the findings. The molecular and immunological alterations observed over time may not reflect patterns consistent across broader patient populations.In addition, the baseline plasma sample was not available. Thus, cytokine profiling before antifungal treatment could not be performed, limiting our ability to fully characterize the patient’s baseline immune status. However, comparisons with healthy controls at subsequent timepoints still revealed marked immune dysregulation. Moreover, whether these immunometabolic alterations are specific to HLH secondary to fungal infection or reflect individual variability remains unclear and warrants further investigation in larger, etiology-stratified cohorts.

In summary, this study highlights the dynamic immunological and metabolic transitions observed in a patient with DH complicated by HLH. With effective antifungal and immunomodulatory treatment, the immune profile evolved from a hyperinflammatory, glycolysis-dominant state toward an oxidative phosphorylation- and lipid metabolism-driven reparative phenotype. These findings suggest that specific, potentially targetable immune signatures may serve as novel diagnostic biomarkers and therapeutic entry points for managing this rare but life-threatening condition.

## Data Availability

The raw data supporting the conclusions of this article will be made available by the authors, without undue reservation.
