# Supplementary material for: Case Report: Longitudinal mass cytometry profiling of a patient with disseminated histoplasmosis and secondary hemophagocytic lymphohistiocytosis
Source: Front Immunol. 2025 Oct 1;16:1660382. doi: 10.3389/fimmu.2025.1660382 (PMC12521218; doi:10.3389/fimmu.2025.1660382)
Supplement: Supplementary file 1 [file Table1.docx]

Supplementary Material 1

**Supplementary Table 1. Details of Literature Searching Strategy**

| Database | Search strategy |
| --- | --- |
| PubMed | (("histoplasmosis"[Mesh] OR "Histoplasma capsulatum"[Title/Abstract])  AND  ("hemophagocytic lymphohistiocytosis"[Mesh] OR "hemophagocytic syndrome"[Title/Abstract] OR "HLH"[Title/Abstract]))  AND  ("2005/01/01"[Date - Publication] : "2025/12/31"[Date - Publication])  AND  (  ("case reports"[Publication Type]  OR "case report"[Title/Abstract]  OR "case series"[Title/Abstract]  OR "clinical study"[Publication Type]  OR "clinical trial"[Publication Type]  OR "observational study"[Publication Type]  OR "cohort"[Title/Abstract]  OR "cross-sectional"[Title/Abstract]  OR "prospective"[Title/Abstract]  OR "retrospective"[Title/Abstract]  OR "laboratory research"[Title/Abstract]  OR "experimental study"[Title/Abstract])  OR  (  ("systematic review"[Publication Type] OR "meta-analysis"[Publication Type])  AND  ("case report"[Title/Abstract] OR "case series"[Title/Abstract]  OR "original data"[Title/Abstract] OR "observational study"[Title/Abstract])  )) |

A total of 34 studies were identified through our literature search. After excluding one study on feline histoplasmosis and two studies without accessible full texts, 31 articles were ultimately included in the analysis. Detailed information on individual cases is summarized in Supplementary Table 2.

**Supplementary Table 2. The Summary of outcomes in patients with histoplasmosis and secondary hemophagocytic lymphohistiocytosis**

| Literature | Country/ Region | Study type | No. of  participants | Gender | Age | Underlying diseases | AIDS | Diagnostic assessment of histoplasmosis | Therapeutic intervention | Outcomes |
| --- | --- | --- | --- | --- | --- | --- | --- | --- | --- | --- |
| Yamasaki L *et al*.,2025 ^(1)^ | Japan | Case Reports | 1 | M | 29 | None | AIDS | BC  SB | AmB/Itra | Recovery |
| Zimmerman *et al*.,2024^(2)^ | USA | Case Reports | 1 | M | 30 | None | AIDS | Urine histoplasma Ag + | AmB/Itra  D | Recovery |
| Jagadish A  *et al*.,2024^(3)^ | USA | Case Reports | 1 | F | 67 | RA | No | Serum/Urine histoplasma Ag +  BM Bx | AmB/Itra | Recovery |
| Chen L  *et al*.,2024^(4)^ | China | Case Reports/ review | 1 | M | 44 | None | No | BM Bx | AmB/Itra | Recovery |
| Alagha Z *et al.*,2024^(5)^ | USA | Case Reports/ review | 1 | F | 57 | RA | No | Serum/Urine histoplasma Ag +  BC | AmB/Itra/Posa  Methylprednisolone | Recovery |
| Akiska YM *et al.*,2024^(6)^ | USA | Case Reports | 1 | M | Adolescent | None | AIDS | Urine histoplasma Ag + | AmB/Itra  Anakinra | Recovery |
| Omo-Ogboi AC *et al*.,2023^(7)^ | USA | Case Reports | 1 | M | 37 | DM,SLE | No | Urine histoplasma Ag +  Peripheral blood smear | NR | Died |
| Tomaino MV *et al*.,2022^(8)^ | Argentina | Case Reports | 1 | M | 32 | leishmaniasis | AIDS | BM Bx | ABD  Methylprednisolone  IVIG | Died |
| Chen H *et al*.,2022^(9)^ | China | Case Reports/Review | 1/13 | M | 46 | None | No | BMC | AmB/Itra  Methylprednisolone  IVIG | Recovery |
| AprianyN *et al.*,2022^(10)^ | Indonesia | Case Reports | 2 | M | 34 | None | No | BMA | voriconazole /Itra | Recovery |
|  |  |  |  | F | 29 | None | No | BMA | voriconazole /Itra | Recovery |
| Kusne Y *et al*.,2021^(11)^ | USA | Case Reports | 2 | F | 66 | RA  chronic low back pain | No | Urine histoplasma Ag +  BM Bx | AmB | Died |
|  |  |  |  | F | 41 | ulcerative colitis | No | Urine histoplasma Ag +  BAL culture | AmB/Itra  High-dose steroids  etoposide | Recovery |
| Castejón-Hernández S *et al*.,2021^(12)^ | Spain | Case Reports | 1 | M | 46 | None | AIDS | BM Bx  PCR | AmB | Died |
| Swaminathan N et al.,2020^(13)^ | USA | Case Reports | 1 | F | 61 | RA | No | BM Bx | AmB | Died |
| Singh S *et al*.,2020^(14)^ | USA | Case Reports | 1 | F | 44 | Ankylosing spondylitis, hypertension,kidney stones, fibromyalgia and obesity | No | Urine histoplasma Ag +  Liver Bx | AmB  DE | Recovery |
| Nguyen D *et al*.,2020^(15)^ | French Guiana | Retrospective study | 14 | M9/F5 | Average age 46 | None | AIDS | BM | AmB[12]/Itra[10]  Glucocorticoid[4]  IVIG[4]  Etoposide[4] | Recovery[12]  Died[2] |
| Tsuboi M *et al.*,2019^(16)^ | Japan | Case Reports | 1 | F | 56 | None | AIDS | Serum/Urine/BM histoplasma Ag +  BMC  PCR | AmB/Itra | Recovery |
| Loganantharaj N *et al*.,2018^(17)^ | The Dominican Republic | Case Reports | 1 | M | 46 | None | AIDS | LN Bx | AmB/Itra | Recovery |
| Ocon AJ *et al*.,2017^(18)^ | French Guiana | Case Reports | 1 | M | 49 | None | AIDS | BM Bx | AmB/Itra  D  Anakinra | Recovery |
| Gómez-Espejo SM *et al*.,2017^(19)^ | Spain | Case Reports | 1 | M | 23 | None | AIDS | Liver Bx | AmB/Itra | Recovery |
| Townsend JL *et al*.,2015^(20)^ | USA | Retrospective study | 11 | M9/F2 | Average age 43.9 | Kidney transplant[1] | AIDS[9] | Urine histoplasma Ag +[9]  BM Bx[8]  BC[7]  Peripheral blood smear[4] | AmB/Itra  Glucocorticoid  IVIG | Recovery[4]  Died[7] |
| Subedee A *et al*.,2015^(21)^ | USA | Case Reports | 1 | F | 42 | None | AIDS | BM Bx  LN Bx | AmB/Itra | Recovery |
| De D *et al*.,2015^(22)^ | India | Retrospective study | 7 | M5/F2 | Average age 35 | None | No | BM Bx[5]  Paranephros Bx[1]  Ulcer swab culture of patients with skin nodules and ulcers[1] | ABD/Itra | Recovery[6]  Died[1] |
| Nieto-Ríos JF *et al*.,2014^(23)^ | USA | Retrospective study | 9 | M4/F5 | 27-54 | Kidney transplant | No | BM Bx [6]  BM[2]  Urine histoplasma Ag +[1] | AmB/Itra[1]  AmB[2]  Itra[5] | Recovery[7]  Died[2] |
| Hegerova LT *et al*.,2013^(24)^ | USA | Case Reports | 1 | M | 41 | Chronic asthma | No | BC  BMC | AmB | Recovery |
| Vaid N et al.,2011^(25)^ | UK | Case Reports | 1 | M | 25 | None | AIDS | BM Bx | Caspofungin | Died |
| Lo MM *et al*.,2010^(26)^ | USA | Case Reports | 2 | F | 22 | Kidney transplant | No | BM Bx | AmB/Itra | Recovery |
|  |  |  |  | M | 18 | Kidney transplant | No | BM Bx | AmB/Itra | Recovery |
| De Lavaissière M *et al*.,2009^(27)^ | France | Case Reports | 1 | M | 33 | None | AIDS | Peripheral blood/BM smear | ABD/Itra  IVIG | Recovery |
| Phillips J  *et al*.,2008^(28)^ | USA | Case Reports | 1 | M | 69 | Sarcoidosis  Prostate cance | No | BMC | AmB/Itra  Corticosteroids  Etoposide  Cyclosporine | Recovery |
| Wang Z *et al*.,2007^(29)^ | USA | Case Reports | 1 | M | 52 | Chronic hepatitis C, hypertension, mixed cryoglobulinemia | No | Autopsy:  Aortic valve neoplasms culture  BC  Spleen culture | None | Died |
| Guiot HM *et al*.,2007^(30)^ | USA | Case Reports | 1 | M | 43 | Ileal perforation | AIDS | Intestinal Bx  BMC  PCR | AmB/Itra | Recovery |
| Gil-Brusola A *et al*.,2007^(31)^ | Spain | Case Reports/ Review | 1/13 | M | 33 | None | AIDS | BC  BMC | None | Died |

F, female; M, male ;NA, not applicable; NR, not reported；+, positive

Bx, biopsy; LN, lymph node; BM, bone marrow; BAL, bronchoalveolar lavage; SB: skin biopsy; BC: blood culture; BMA :bone marrow aspiration; BMC:bone marrow culture;

RA, rheumatoid arthritis;DM, diabetes-mellitus;

AmB, amphotericin B; Itra, itraconazole; Posa, posaconazole; D, dexamethasone ; DE,IVIG, intravenous immunoglobulin; ABD, amphotericin B deoxycholate；

[ ]，numbers within the square brackets correspond to the number of cases involved in the research.

**References**

1. Yamasaki L, Akiyama Y, Ueno K, Hoshino Y, Nagi M, Nakayama N, et al. Progressive Severe Hemophagocytic Syndrome due to Disseminated Histoplasmosis in a Patient with HIV-1 Infection. *Intern Med* (2025) 64(7):1113-8. doi: 10.2169/internalmedicine.4079-24

2. Zimmerman JT, Hanson C, Iardino A. Haemophagocytic lymphohistiocytosis (HLH) secondary to disseminated histoplasmosis infection in a patient with HIV. *BMJ Case Rep* (2024) 17(8):e259484 [pii]. doi: 10.1136/bcr-2023-259484

3. Jagadish A, Notta SN, Notta N, Raafey MA, Falasca G. Disseminated Histoplasmosis Mimicking Macrophage Activation Syndrome in a Patient With Rheumatoid Arthritis. *Cureus* (2024) 16(2):e53723. doi: 10.7759/cureus.53723

4. Chen L, Hu D, Zhang C, Wu T, Cheng X, Hagen F, et al. Histoplasmosis: An epidemiological and clinical update in China, review and a case report. *Mycology* (2024) 15(1):101-9. doi: 10.1080/21501203.2023.2259934

5. Alagha Z, Bills E, Al-Hiari M, Abdeen AM, Zeid F. Survival Against the Odds-Hemophagocytic Lymphohistiocytosis Amidst the Shadows of Disseminated Histoplasmosis: A Case Report and Literature Review. *J Investig Med High Impact Case Rep* (2024) 12:23247096241258074. doi: 10.1177/23247096241258074

6. Akiska YM, Koay W, Unternaher J, Rakhmanina NY. Successful management of haemophagocytic lymphohistiocytosis in an adolescent with newly diagnosed HIV/AIDS and histoplasmosis. *BMJ Case Rep* (2024) 17(6):e260060 [pii]. doi: 10.1136/bcr-2024-260060

7. Omo-Ogboi AC, Shirai S, Ur Rehman A, Ederhion JO, Buja M. A Rare Case of Disseminated Histoplasmosis With Hemophagocytic Lymphohistiocytosis Mimicking a Flare of Systemic Lupus Erythematosus in a Middle-Aged Man: A Case Report. *Cureus* (2023) 15(9):e46068. doi: 10.7759/cureus.46068

8. Tomaino MV, Barletta JA, Andreani M, Sisto A, Abusamra L, Guelfand L, et al. Hemophagocytic lymphohistiocytosis secondary to AIDS-related histoplasmosis and visceral leishmaniasis. *Int J STD AIDS* (2022) 33(9):873-6. doi: 10.1177/09564624221110986

9. Chen H, Yuan Q, Hu H, Wang J, Yu M, Yang Q, et al. Hemophagocytic Lymphohistiocytosis Secondary to Disseminated Histoplasmosis in HIV Seronegative Patients: A Case Report and Review of the Literature. *Front Cell Infect Microbiol* (2022) 12:847950. doi: 10.3389/fcimb.2022.847950

10. Apriany N, Sukorini U, Ratnaningsih T, Asdie RH, Subronto YW, Hutajulu SH, et al. Two Cases of Hemophagocytic Lymphohistiocytosis Associated with Disseminated Histoplasmosis Presented with Transient Pancytopenia. *Case Rep Med* (2022) 2022:9521128. doi: 10.1155/2022/9521128

11. Kusne Y, Christiansen M, Conley C, Gea-Banacloche J, Sen A. Hemophagocytic Lymphohistiocytosis Secondary to Disseminated Histoplasmosis in Rheumatologic Disease. *Case Rep Crit Care* (2021) 2021:6612710. doi: 10.1155/2021/6612710

12. Castejón-Hernández S, Reynaga-Sosa EA, Navarro-Aguirre M, Vilamala-Bastarras A. Hemophagocytic lymphohistiocytosis (HLH) caused by disseminated histoplasmosis by H. capsulatum var. duboisii in HIV patient: A case report. *Enferm Infecc Microbiol Clin (Engl Ed)* (2021) 39(2):102-3. doi: 10.1016/j.eimc.2020.04.001

13. Swaminathan N, Vinicius JM, Serrins J. Hemophagocytic Lymphohistiocytosis (HLH) in a Patient with Disseminated Histoplasmosis. *Case Rep Hematol* (2020) 2020:5638262. doi: 10.1155/2020/5638262

14. Singh S, Thanikachalam K, Donthireddy V. Desperate times, desperate measures: successful use of chemotherapy in treatment of haemophagocytic lymphohistiocytosis (HLH) due to disseminated histoplasmosis. *BMJ Case Rep* (2020) 13(9):e235144. doi: 10.1136/bcr-2020-235144

15. Nguyen D, Nacher M, Epelboin L, Melzani A, Demar M, Blanchet D, et al. Hemophagocytic Lymphohistiocytosis During HIV Infection in Cayenne Hospital 2012-2015: First Think Histoplasmosis. *Front Cell Infect Microbiol* (2020) 10:574584. doi: 10.3389/fcimb.2020.574584

16. Tsuboi M, Nishijima T, Nagi M, Miyazaki Y, Teruya K, Kikuchi Y, et al. Case Report: Hemophagocytic Lymphohistiocytosis Caused by Disseminated Histoplasmosis in a Venezuelan Patient with HIV and Epstein-Barr Virus Reactivation Who Traveled to Japan. *Am J Trop Med Hyg* (2019) 100(2):365-7. doi: 10.4269/ajtmh.18-0478

17. Loganantharaj N, Oliver B, Smith T, Jetly R, Engel L, Sanne S. Hemophagocytic lymphohistiocytosis in an HIV-positive patient with concomitant disseminated histoplasmosis. *Int J STD AIDS* (2018) 29(9):925-8. doi: 10.1177/0956462417753008

18. Ocon AJ, Bhatt BD, Miller C, Peredo RA. Safe usage of anakinra and dexamethasone to treat refractory hemophagocytic lymphohistiocytosis secondary to acute disseminated histoplasmosis in a patient with HIV/AIDS. *BMJ Case Rep* (2017) 2017:bcr2017221264. doi: 10.1136/bcr-2017-221264

19. Gómez-Espejo SM, Olalla-Sierra J, Marí-Jiménez P, Pereda-Salguero T, Pérez-Stachowski J, de-la-Torre-Lima J, et al. Reconstitution Inflammatory Syndrome Like Reactive Hemophagocytic Syndrome Associated with Disseminated Histoplasmosis in a HIV Patient. *Mycopathologia* (2017) 182(7-8):767-70. doi: 10.1007/s11046-017-0144-6

20. Townsend JL, Shanbhag S, Hancock J, Bowman K, Nijhawan AE. Histoplasmosis-Induced Hemophagocytic Syndrome: A Case Series and Review of the Literature. *Open Forum Infect Dis* (2015) 2(2):ofv055. doi: 10.1093/ofid/ofv055

21. Subedee A, Van Sickels N. Hemophagocytic Syndrome in the Setting of AIDS and Disseminated Histoplasmosis: Case Report and a Review of Literature. *J Int Assoc Provid AIDS Care* (2015) 14(5):391-7. doi: 10.1177/2325957415570740

22. De D, Nath UK. Disseminated Histoplasmosis in Immunocompetent Individuals- not a so Rare Entity, in India. *Mediterr J Hematol Infect Dis* (2015) 7(1):e2015028. doi: 10.4084/MJHID.2015.028

23. Nieto-Ríos JF, Serna-Higuita LM, Guzman-Luna CE, Ocampo-Kohn C, Aristizabal-Alzate A, Ramírez I, et al. Histoplasmosis in renal transplant patients in an endemic area at a reference hospital in Medellin, Colombia. *Transplant Proc* (2014) 46(9):3004-9. doi: 10.1016/j.transproceed.2014.06.060

24. Hegerova LT, Lin Y. Disseminated histoplasmosis: a cause of hemophagocytic syndrome. *Mayo Clin Proc* (2013) 88(10):e123. doi: 10.1016/j.mayocp.2013.04.030

25. Vaid N, Patel P. A case of haemophagocytic syndrome in HIV-associated disseminated histoplasmosis. *Acute Med* (2011) 10(3):142-4.

26. Lo MM, Mo JQ, Dixon BP, Czech KA. Disseminated histoplasmosis associated with hemophagocytic lymphohistiocytosis in kidney transplant recipients. *Am J Transplant* (2010) 10(3):687-91. doi: 10.1111/j.1600-6143.2009.02969.x

27. De Lavaissière M, Manceron V, Bourée P, Garçon L, Bisaro F, Delfraissy JF, et al. Reconstitution inflammatory syndrome related to histoplasmosis, with a hemophagocytic syndrome in HIV infection. *J Infect* (2009) 58(3):245-7. doi: 10.1016/j.jinf.2008.11.010

28. Phillips J, Staszewski H, Garrison M. Successful treatment of secondary hemophagocytic lymphohistiocytosis in a patient with disseminated histoplasmosis. *Hematology* (2008) 13(5):282-5. doi: 10.1179/102453308X316013

29. Wang Z, Duarte AG, Schnadig VJ. Fatal reactive hemophagocytosis related to disseminated histoplasmosis with endocarditis: an unusual case diagnosed at autopsy. *South Med J* (2007) 100(2):208-11. doi: 10.1097/SMJ.0b013e31802b2812

30. Guiot HM, Bertrán-Pasarell J, Tormos LM, González-Keelan C, Procop GW, Fradera J, et al. Ileal perforation and reactive hemophagocytic syndrome in a patient with disseminated histoplasmosis: the role of the real-time polymerase chain reaction in the diagnosis and successful treatment with amphotericin B lipid complex. *Diagn Microbiol Infect Dis* (2007) 57(4):429-33. doi: 10.1016/j.diagmicrobio.2006.09.010

31. Gil-Brusola A, Pemán J, Santos M, Salavert M, Lacruz J, Gobernado M. Disseminated histoplasmosis with hemophagocytic syndrome in a patient with AIDS: description of one case and review of the Spanish literature. *Rev Iberoam Micol* (2007) 24(4):312-6. doi: 10.1016/s1130-1406(07)70063-3

**
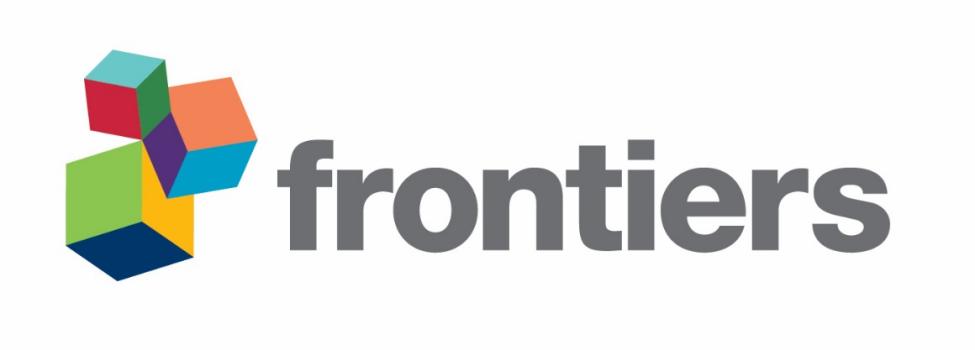
**
