# Supplementary material for: Case Report: Longitudinal mass cytometry profiling of a patient with disseminated histoplasmosis and secondary hemophagocytic lymphohistiocytosis
Source: Front Immunol. 2025 Oct 1;16:1660382. doi: 10.3389/fimmu.2025.1660382 (PMC12521218; doi:10.3389/fimmu.2025.1660382)
Supplement: Supplementary file 2 [file Table2.docx]

Supplementary Material 2

**Materials and Methods**

## Isolation of PBMC

PBMC were isolated by Histopaque-based (10771; Sigma-Aldrich, St Louis, MO, USA) density gradient centrifugation. Thereafter, PBMC were lysed in 2 ml of red blood cell lysis buffer at −80°C (Solarbio, BJ, CN). Cell viability was assessed with trypan blue (Solarbio, BJ, CN).

## Flow cytometry

PBMC were stained with anti-human CD4 (BD Biosciences, USA) for flow cytometric analysis (FACSCanto II and LSRFortessa, BD Biosciences, USA).

## Cell labelling and mass cytometry flow analysis

As previously described(1), PBMC samples were labelled with cisplatin-195Pt and quenched with Cell Staining Buffer (Fluidigm) for viability assessment. Cells from multiple samples were probed with a CD45 barcode to minimize inter-sample staining variation. After Fc-receptor blocking, cells were directly incubated with metal-labelled antibodies in Cell Staining Buffer, without methanol permeabilization, and were then fixed with paraformaldehyde. DNA was labelled with a DNA intercalator. The mass-cytometry antibody panel design is shown in Supplementary Table 3. After washing three times with double-distilled water, cells were resuspended in collection buffer containing 10 % EQ beads (Fluidigm, CA, USA) for detection (Helios mass cytometry, USA).

The original flow cytometry standard data were normalized. The standardized flow cytometry data were uploaded to Cytobank (<https://www.cytobank.org/>), in which cell debris and doublets were removed and living cells were gated. Cells were clustered based on their expression levels of cell surface proteins and the results were visualized. The living cells were gated based on cisplatin labelling signals, and the results were analysed with R and PhenoGraph (<https://bioconductor.org/packages/cytofkit/>).

## Serum biomarkers

Fasting plasma samples were collected from healthy controls and PLWH before and after their third rounds of vaccination. The circulating levels of cytokines and chemokines at day 14 after vaccination were determined with a convenient Bioplex assay kit (LINCO Research) according to the manual. The inflammatory molecules included sCD40L、EGF、Eotaxin、FGF-2、FLT-3L、fractalkine、G-CSF、GM-CSF、GROα、IFNϒ、IL-1α、IL-1β、IL-2、IL-3、IL-4、IL-5、IL-6、IL-7、IL-8、IL-9、IL-10、IL-12（p40）、IL-12（p70）、IL-13、IL-15、IL-17A、IL-17E/IL-25、IL-17F、IL-18、IL-22、IL-27、IP-10、MCP-1、MCP-3、M-CSF、MDC、MIG、MIP-1α、MIP-1β、PDGF-AA、PDGF-AB/BB、RANTES、TGFα、TNFα、TNFβ、VEGF-A。

**Supplementary table 3.** **Mass cytometry antibodies panel design.**

| Antigen | Symbol and Mass | Antibody clone | Source |
| --- | --- | --- | --- |
| 142Ce | IFNG | EPR23991-53 | abcam |
| 176Lu | CD68 | BL13756 | POLARIS |
| 153Eu | CPT1A | EPR21843-71-1C | abcam |
| 165Ho | CD36 | 255606 | R＆D |
| 151Eu | CD11B | ICRF44 | Fluidigm |
| 146Nd | CD8 | 37006 | R＆D |
| 145Nd | GLUT1 | SP168 | abcam |
| 144Nd | CD20 | 2H7 | Fluidigm |
| 150Sm | SDH | EPR9043(B) | abcam |
| 149Sm | CD25 | 2A3 | Fluidigm |
| 148Sm | MTOR | EPR427(N) | abcam |
| 158Gd | IDH | EP1565Y | abcam |
| 156Gd | PGC1A | 4A8 | abcam |
| 155Gd | PD_1 | 913429 | R＆D |
| 164Dy | CD98 | MEM-108 | BioLegend |
| 161Dy | CD206 | 2月15日 | POLARIS |
| 163Dy | IL_10 | EPR1114 | abcam |
| 162Dy | CD11C | Bu15 | Fluidigm |
| 160Dy | CD19 | HIB19 | Fluidigm |
| 166Er | CD56 | 301021 | R＆D |
| 168Er | CD4 | 34930 | R＆D |
| 167Er | CCR7 | G043H7 | Fluidigm |
| 172Yb | CD57 | REA769 | POLARIS |
| 173Yb | HLA_DR | L243 | POLARIS |
| 170Yb | IL_17 | QA18A46 | BioLegend |
| 154Gd | LDH | EP1565Y | abcam |
| 147Sm | CD45RO | UCHL1 | BioLegend |
| 143Nd | CD45RA | HI100 | Fluidigm |
| 169Tm | CD14 | 134620 | R＆D |
| 159Tb | CD86 | 37301 | R＆D |
| 152Gd | TNFA | EPR20972 | abcam |
| 141Pr | PKM2 | EPR10138(B) | abcam |
| 209Bi | CD3 | UCHT-1 | POLARIS |
| 89Y | CD45 | HI30 | Fluidigm |

1. Zhang J, Yin J, Wang W, Gao J, Li T, Mou D, et al. Reduced Neutralizing Antibody Production against Omicron Xbb.1.5 in People Living with Hiv. *Chin Med J (Engl)* (2024) 137(21):2633-5. doi: 10.1097/CM9.0000000000003139.

**
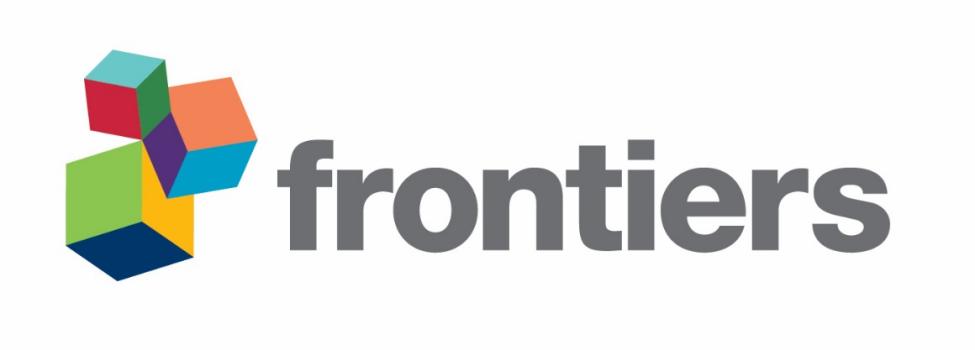
**
